# Blood-testis barrier integrity depends on Pin1 expression in Sertoli cells

**DOI:** 10.1038/s41598-017-07229-1

**Published:** 2017-08-01

**Authors:** Rabia Islam, Heein Yoon, Bong-soo Kim, Han-sol Bae, Hye-rim Shin, Woo-Jin Kim, Won-joon Yoon, Yun-Sil Lee, Kyung Mi Woo, Jeong-Hwa Baek, Hyun-Mo Ryoo

**Affiliations:** Department of Molecular Genetics, School of Dentistry and Dental Research Institute, Seoul National University, Seoul, 110-749 Korea

## Abstract

The conformation and function of a subset of serine and threonine-phosphorylated proteins are regulated by the prolyl isomerase Pin1 through isomerization of phosphorylated Ser/Thr-Pro bonds. Pin1 is intensely expressed in Sertoli cells, but its function in this post mitotic cell remains unclear. Our aim was to investigate the role of Pin1 in the Sertoli cells. Lack of Pin1 caused disruption of the blood-testis barrier. We next investigated if the activin pathways in the Sertoli cells were affected by lack of Pin1 through immunostaining for Smad3 protein in testis tissue. Indeed, lack of Pin1 caused reduced Smad3 expression in the testis tissue, as well as a reduction in the level of N-Cadherin, a known target of Smad3. Pin1^−/−^ testes express Sertoli cell marker mRNAs in a pattern similar to that seen in Smad3^+/−^ mice, except for an increase in Wt1 expression. The resulting dysregulation of N-Cadherin, connexin 43, and Wt1 targets caused by lack of Pin1 might affect the mesenchymal–epithelial balance in the Sertoli cells and perturb the blood-testis barrier. The effect of Pin1 dosage in Sertoli cells might be useful in the study of toxicant-mediated infertility, gonadal cancer, and for designing male contraceptives.

## Introduction

The testis is an immune-privileged organ that protects itself from auto-antigens and the associated detrimental immune responses by forming a blood-testis barrier (BTB)^1^. Infertility is a common problem affecting almost one in six couples, with 50% of cases attributed to male infertility resulting from abnormalities, of which 60–75% are found to be idiopathic^2, 3^. Sertoli cells (SCs) have historically been called the testicular ‘nurse cells,’ and the proper organization and maturation of the Sertoli cell population underpin adult male fertility.

Testicular Sertoli cells play important roles in spermatogenesis as they nourish sperm cells and contribute to the formation of the BTB that plays a critical role in the physiology and pathology of the testes in mammals^4, 5^. SCs are specialized polarized epithelial cells that extend from the base of the seminiferous tubule to its lumen. SCs are the first somatic cells to differentiate in the testes and are thought to direct further testes development^6^. Factors affecting blood-testis barrier function might be involved in testicular damage and male infertility. During the seminiferous epithelial cycle of spermatogenesis in the mammalian testis, multiple cellular events take place across the seminiferous epithelium, including spermatogonial self-renewal via mitosis, meiosis, spermiogenesis, and spermiation, all of which are supported by SCs^5, 7^. In particular, SCs produce numerous factors, such as glial cell line-derived neurotrophic factor (GDNF), fibroblast growth factor 2 (FGF2), bone morphogenic protein 4 (BMP4), and stem cell factor (SCF), which initiate the differentiation of spermatogonial stem cells (SSCs)^8–11^.

Sertoli cells create a local tolerogenic microenvironment to maintain testicular immune privilege especially through the formation of the BTB, which separates the inner tubular microenvironment from the rest of the body^1^. In mammals, the BTB is created by adjacent Sertoli cells in the seminiferous epithelium near the basement membrane via coexisting specialized tight junction (TJ), basal ectoplasmic specialization (ES, a testis-specific atypical adherens junction [AJ] type), and desmosome-like junctions. Previous studies have identified several integral membrane protein complexes, such as the occludin–ZO-1 complex at the TJ and the N-cadherin–β-catenin complex at the basal ES, that constitute the BTB and are irreplaceable for the maintenance of the BTB in mammalian testes. The regulatory proteins that control the Sertoli cell permeability barrier remain mostly unknown. This information would be of considerable use to investigators in the field of infertility and gonadal cancer. The BTB, unlike other blood–tissue barriers such as the blood–brain barrier, is not a static barrier because it must restructure to allow the passage of primary spermatocytes while maintaining the immunological barrier to protect post-meiotic germ cell development from systemic circulation and resist production of auto antigens. The BTB confers a barrier function to regulate the passage of biomolecules, water, hormones, and other substances from the basal to the adluminal compartment. Disruption of barrier function and integrity (by environmental toxicants such as bisphenol A and cadmium or radiation) leads to testicular injury and infertility^7, 12^. Regulation of the BTB could also be the key to the development of much needed male contraceptives.

BTB integrity is thought to be associated with testicular dysgenesis syndrome^13^ and the unexplained male infertility accounting for 30–40% of men with abnormal semen parameters^14^.

Pin1 (peptidylprolyl cis/trans isomerase, NIMA-interacting 1) is a peptidyl-prolyl cis/trans isomerase (PPIase) that specifically catalyzes the cis/trans isomerization of peptidyl-prolyl peptide bonds, preceded by a phosphorylated serine or threonine residue. Pin1 shows higher PPIase activity in brain and testis compared to other tissues like lungs or liver^15^. The basal activity by other PPIases cannot sufficiently replace Pin1 deficiency in these organs, and that was assumed to be the reason Pin1^−/−^ mice show phenotypes with prominent differences in the brain and testis^15^. Immunostaining of Pin1 in a wild-type testis resulted in ubiquitous Pin1 expression in the seminiferous tubules. However, more intense Pin1 expression is seen along the periphery of the tubule. It is most highly expressed in spermatogonia and Sertoli cells^16^. Although the role of Pin1 in germ cells has been extensively studied, the role of Pin1 in Sertoli cells has not been reported. In germ cells, the existing knowledge cannot explain the wide spread gonadal dysgenesis and infertility seen in Pin1^−/−^ mice^16, 17^. Pin1 was also investigated as one of the main mediators of testicular injury during heavy ion radiation (HIR) on mouse testes^18^. Previously, we reported that Pin1 has a role in cell-to-cell fusion in osteoclasts^19, 20^. BTB formation also depends on cell-to-cell interactions^5, 21^ and might be an important target site for Pin1-mediated regulation in a cell biological context and might also shed some light on the function of Pin1 in a post mitotic cell. Even though Pin1 is expressed in Sertoli cells, consistent with its prospective role in cell-to-cell contact at the BTB, there is no existing literature on this topic. We sought to investigate whether Pin1 is an integral component of Sertoli cell physiology and to elucidate its role in maintenance of the BTB.

## Materials and Methods

### Ethics statement and animals

The animal experiments were performed according to the Guide for the Care and Use of Laboratory Animals, and all experimental protocols were approved by the Animal Care and Use Committee of Seoul National University under animal protocol number SNU-120327-6-3. Homozygous Pin1-mutant mice, used previously in our laboratory, were obtained from Professor Takafumi Uchida of Tohuko University^22^. The Pin1 gene deletion was transferred into an isogenic C57BL/6 J background with 20 breeding cycles. All mice were maintained on a C57BL/6 J background. BrdU was diluted in PBS to make a sterile solution of 10 mg/mL. For mice, the BrdU solution at a concentration of 100 mg/kg was injected 3 Hr before sacrifice.

### Sertoli cell isolation and Western blotting

Mouse Sertoli cells were isolated from 4-week-old mice using previously reported protocols^23, 24^. Testes from 5 mice were aseptically removed, decapsulated, minced, and washed twice with sterile PBS. The minced tissues were then digested with 0.25% trypsin at 37 °C for 6 min, followed by digestion in 0.1% collagenase I at 37 °C for 10 min. Next, the obtained homogenate was filtered through a 150-μm filter, and cells were collected by centrifugation at 1000 RPM for 5 min. After being washed with PBS 3 times, the isolated Sertoli cells were re-suspended in culture medium containing 90% DMEM medium and 10% FBS and then plated on cell culture dishes. Next, these cultures were subjected to a hypotonic treatment to lyse residual germ cells. Virtually all contaminating germ cells were lysed via a brief hypotonic treatment using 20 mM Tris, pH 7.4 at 22 °C for 2.5 min at room temperature. Immediately after hypotonic shock application, the purified Sertoli cells were pelleted and homogenized in 500 ml ice-cold RIPA buffer (50 mM Tris-HCL PH 7.4, 150 mM NaCl, 1% NP, 0.1% SDS, 1 × phosphatase/protease inhibitors) to extract proteins. The protein concentration was determined using the BCA protein assay kit (Beyotime, Nantong, China). About 20 μg of protein from each sample was separated by 10% SDS-PAGE and electrophoretically transferred to a polyvinylidene fluoride (PVDF) membrane. The membrane was then blocked in TBS buffer containing 5% bovine serum albumin (20 mM Tris–HCl [pH 7.6] and 150 mM NaCl) for 1 h at 37 °C. Blots were then incubated overnight at 4 °C with anti Smad3 (Smad3 antibody number 9513, Cell Signaling Technologies) antibody and ß-actin antibody, followed by HRP-conjugated secondary antibody. Blots were imaged ﻿(Supplementary Fig. 1) using the GeneGnome XRQ system (Syngene, Seoul, Korea)

### Cell culture

The mouse Sertoli cell line TM4 was obtained from the Korean Cell Line Bank, Korean Cell Line Research Foundation and cultured according to standard protocols. After subculturing the murine Sertoli-like cell line TM4 cells were cultured in medium consisting of 50% DMEM and 50% F-12 medium containing 2.5% FBS or 5% horse serum, 14 mM NaHCO3, and 15 mM HEPES in a humidified incubator at 37 °C under 5% CO2.

### qRT-PCR validation analyses of target genes

Total RNA from the testes and Sertoli cells was isolated using Trizol reagent (Invitrogen, Thermo Fisher Scientific, Waltham, MA, USA). To examine mRNA levels, 1 μg of total RNA was reverse transcribed using a first strand cDNA synthesis kit (Takara, Japan). qRT-PCR was conducted using the SYBR Green qRT-PCR kit (Takara) on an Applied Biosystems 2500 Real-Time PCR machine (Applied Biosystems). qRT-PCR was carried out in a 20 μl reaction mixture, which consisted of 10 μl of 2 × SYBR Green Mix, 0.2 μl of forward and reverse primers (20 μM of each primer), 0.4 μl of ROXII dye, and 2 μl diluted cDNA. Cycling conditions were denaturation at 95 °C for 30 s, followed by 40 cycles of denaturation for 15 s at 95 °C, annealing for 34 s at 60 °C, and extension for 30 s at 72 °C, followed by a melting step.

### Immunohistochemistry and immunohistofluorescence

Testes and epididymides were dissected and fixed in Bouin’s fixative overnight at 4 °C. The fixed tissues were washed several times in ice-cold 70% ethanol and paraffin embedded. One testis was fixed in Bouin’s fixative and sectioned at approximately 5-μm thickness onto slides. The other testis was snap frozen on dry ice and then stored at −80 °C. Immunohistochemistry was then performed. For histology, tissue samples were stained with hematoxylin and eosin, as well as periodic acid-Schiff reagent and hematoxylin (PAS-H) (PolyScientific, Bay Shore, NY). Briefly, sections were deparaffinized and rehydrated, followed by antigen retrieval in 10 mM sodium citrate buffer. Antigen retrieval was achieved using a CPC-600 pressure cooker (Cuisinart, NJ, USA) by heating samples using the low pressure setting for 10 min in citrate buffer and then cooling for 20 min [Antibodies used were SMAD3 (Smad3 Antibody number 9513, Cell Signaling Technology), Connexin 43 (Connexin 43 Antibody number 3512, Cell Signaling Technology), Br Du (Anti-BrdU clone B44, Catalogue number 347580), N-cadherin (Anti N-cadherin clone H-63, sc-7939, Santa cruz) and γH2AX/ Phospho-Histone H2A.X (Ser139/Tyr142) Antibody #5438]. Bound primary antibodies were detected using biotinylated anti-rabbit antibody (Vector Laboratories, Burlingame, CA, USA) or biotinylated anti-mouse (Vector Laboratories). Signal was amplified with Vectastain Elite ABC kit reagents (Vector Laboratories) according to the manufacturer’s instructions and detected with 3,3-diaminobenzidine tetrahydrochloride (Dako). Nuclei were visualized using Harris hematoxylin counterstaining. Sections were dehydrated in an ethanol series and mounted under DPX (BDH Laboratories, Poole, UK). Immunohistochemistry was performed on at least two sections more than 200 μm apart from at least three independent animals per genotype per age. For each experiment, determination of nonspecific binding of secondary antibodies was evaluated simultaneously ﻿(Supplementary Fig. 2). Immunofluorescence analyses of testicular tissues were performed as previously described^19^. Testis cross-sections were fixed with ice-cold methanol and permeabilized with 0.1% Triton X-100 (vol/vol). Primary antibodies were diluted 1:100 in 1% BSA (wt/vol), and cross-sections were incubated overnight at room temperature with primary antibodies. After a brief washing step, cross-sections were exposed for 30 minutes to fluorescent dye-conjugated secondary antibodies (Jackson ImmunoResearch Inc., West Grove, PA, USA). Sections were mounted with DAPI-containing mounting media (VECTASHIELD® Mounting Medium with DAPI. Catalog No. H-1200, Vector Laboratories).

### Biotin-tracer assay

The permeability of the BTB was assessed using EZ-Link Sulfo-NHS-LC-Biotin (Pierce Chemical Co., Grand Island, NY, USA). Twenty microliters of fresh PBS solution containing 10 mg/ml biotin and 1 mM CaCl2 was injected into the interstitium of the testes in 4-week-old mice. The animals were euthanized 30 min later, and the testes were immediately removed and embedded in OCT tissue freezing medium (Leica Biosystems, IL, USA). Fluorescence images of the cryosections were analyzed after staining with a streptavidin reagent conjugated with Cy3 (SA1010, Thermo Fisher Scientific, Waltham, MA, USA) and captured with confocal laser scanning microscopy (LSM 700, Carl Zeiss).

### Dextran FITC injection and BTB assay

Fluorescein isothiocyanate – Dextran 500000-Conjugate (Cat# 46947, Sigma Aldrich, USA) was injected through the jugular vein; 2 hr later, the mice were sacrificed. Testicular tissue was fixed immediately after sacrifice and paraffin embedded. Five-μm-thick sections were deparaffinized and mounted with DAPI-containing mounting solution (Vector Laboratories).

### Statistics

The experiments were repeated at least three times. For immunohistochemistry and immunofluorescence experiments, one representative image from the results of three independent experiments is presented. The data were evaluated for significant differences using Student’s t-test. A P value < 0.05 was considered significant.

## Results

### Pin^−/−^ mice demonstrate subfertility in a C57BL6 background

Several previous studies found that Pin1^−/−^ mice are generally infertile at breeding age^25, 26^. However, some reports have stated that Pin1^−/−^ female and male mice were fertile; nevertheless, the success rate of homozygous cross breeding was much lower or took much longer than that of wild-type or heterozygous mice^17, 27^. This difference was attributed to genetic background, as on a mixed genetic background, the Pin1^−/−^ males and females retained reproductive capability and displayed normal reproductive organ morphology until older males developed mild testicular atrophy. However, when transferred into an inbred C57BL/6 J background, mated Pin1^−/−^ males and females failed to reproduce a single offspring^17, 27, 28^. To confirm the phenotype in our setting with an inbred strain in the C57Bl6 background, we evaluated the impact of Pin1 deletion on male fertility using a successive breeding assay. To assay the generative ability, 10-week-old Pin1^−/−^ male mice or littermate controls were mated with wild type female mice. The observation of a vaginal plug was not different between the two groups of mice (data not shown). One week later, the male mice were again mated with different wild type female mice. These mating experiments were repeated twice successively. After a total of 18 matings, Pin1^+/+^ mice sired a total of 183 progenies, whereas the Pin1^−/−^ male mice produced only one litter with 9 progeny born from a single pregnant female after 19.5 days of gestation (Table 1). We mated 3 Pin1^−/−^ male mice with 3 Pin1^+/+^ female mice each, and the experiment was repeated 2 times with an expected total of 18 copulation events according to vaginal plug observations. Our results confirmed that, although Pin1^−/−^ mice in our experimental model are sub-fertile as adults, they are not infertile.

**Table 1:** The generative ability of transgenic male mice was evaluated by 2 successive matings with wild type female mice (3 male Pin1^+/+^ or Pin1^−/−^ mice with 3 female Pin1^+/+^ each, repeated 2 times).

Genotype (male)	Number of Litters	Total number of pups	Average litter size
Pin1 +/+ (n = 18)	17	183	10.7 ± 1.89
Pin1−/− (n = 18)	1	9	N/A

### Pin^−/−^ mice show almost normal seminiferous epithelium at 4 weeks of age and then gradual lose germ cells

**Figure 1:**
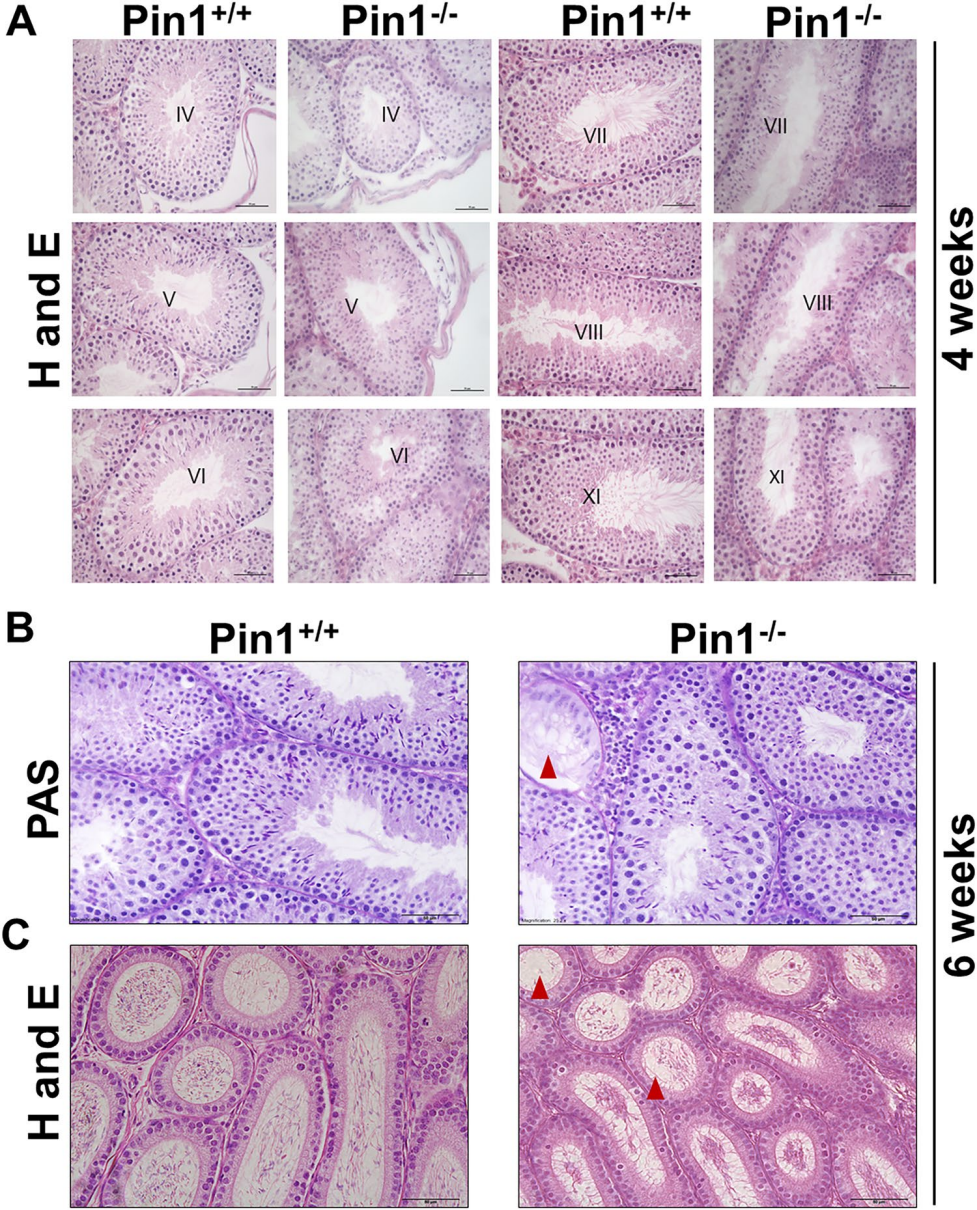
Pin1 ablation causes abnormal seminiferous epithelium and a gradual loss of germ cells. (**A**) The histological structure of seminiferous epithelium stained with hematoxylin and eosin (H and E) in Pin1^+/+^ and Pin1^−/−^ mice at 4 weeks of age (scale bar = 50 µM). (**B**) Periodic acid–Schiff (PAS) stain of histological sections from 6-week-old Pin1^+/+^ and Pin1^−/−^ mice. Arrowheads indicate abnormal phenotypes of the seminiferous epithelium including vacuolation. (**C**) H and E-stained sections of the epididymides of 6-week-old mice (scale bar = 50 µM).

We observed that, by late adulthood (3 months of age), the male knockout mice were infertile, and that the testes were small and exhibited a damaged structure, as reported previously^17^. Testicular atrophy was previously observed in Pin1^−/−^ mice at 3.5 months of age, and by 3–5 months of age, the average weight of Pin1^−/−^ testes was only 56% of that of wild-type mice^17^. At 4 weeks of age, our Pin1^−/−^ testis analysis revealed that all seminiferous tubules contained germ cells organized in the multi-layered epithelium. Spermatogonia, spermatocytes, and spermatids produced during the normal process of spermatogenesis were clearly identified (Fig. 1A). In agreement with previous reports, we also found that the Pin1^−/−^ testis contained tubules with multilayered seminiferous epithelia, including stage VIII tubules with mature spermatids lining the lumen (Fig. 1A). This indicated that Pin1^−/−^ germ cells were able to complete the spermatogenic process and produce normal sperm. However, by 6 weeks of age, seminiferous tubules with vacuoles were observed (Fig. 1B). Many Sertoli cells (SCs) exhibited abnormal features, such as rounded nuclei and absence of tubular lumina and cytoplasmic vacuoles. They also exhibited areas with Sertoli cells lacking germ cells. Pin1^−/−^ testis showed many such Sertoli cell-only tubules. Compared to wild type mice at 6 weeks of age, the corresponding sections of epididymis in Pin1^−/−^ mice showed few mature sperm, confirming azoospermia (Fig. 1C). The degeneration occurred progressively in Pin1^−/−^ testis in an age-dependent manner and started after 4 weeks of age, which is after the Sertoli cell maturation period.

### Meiosis and proliferation are unhampered in Pin1^−/−^ testis

**Figure 2:**
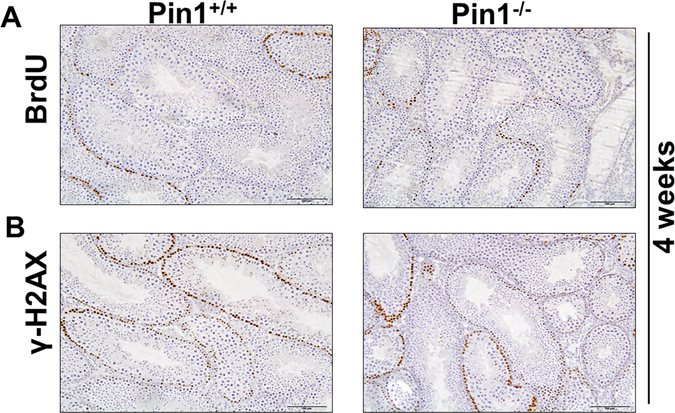
Loss of Pin1 does not hamper progression of meiosis or proliferation. (**A**) BrdU staining was observed in Pin1−/− mouse testicular tissue at 4 weeks of age. Mice were injected with BrdU (100 µg/gram body weight) 12 hours prior to sacrifice. (**B**) γ-H2AX immunostaining was observed in the same tissue to analyze the progression of meiosis (scale bar = 100 µM).

Along the BTB, tight junctions coexist and cofunction with ectoplasmic specializations, desmosomes, and gap junctions to create a unique microenvironment for the completion of meiosis and the subsequent development of spermatids into spermatozoa via spermiogenesis. We stained testicular tissue from 4-week-old mice with BrdU and γ-H2AX, a meiotic marker, and found that the staining pattern was near normal, confirming that proliferation and meiosis were proceeding in Pin^−/−^ testes. BrdU staining was not highly reduced as expected (Fig. 2A) because of the role of Pin1 as a cell cycle protein. Previous reports also confirmed that the percentage of Pin1^−/−^ primordial germ cells in mitosis (6.4%) was not significantly different in wild type (5.8%), indicating that mammalian Pin1-deficient PGCs progressed through mitosis^27^. Likely, there is a small change that was not apparent in our immunostained samples with BrdU staining. The γ-H2AX staining pattern was similar to that of wild type tissue, except for some highly stained cords that did not show a lumen (Fig. 2B). Overall, we observed that both proliferation and meiosis were progressing in Pin^−/−^ testis at 4 weeks of age.

### Pin1^−/−^ epididymis has blood cells inside the lumen and increased apoptosis in the testis

**Figure 3:**
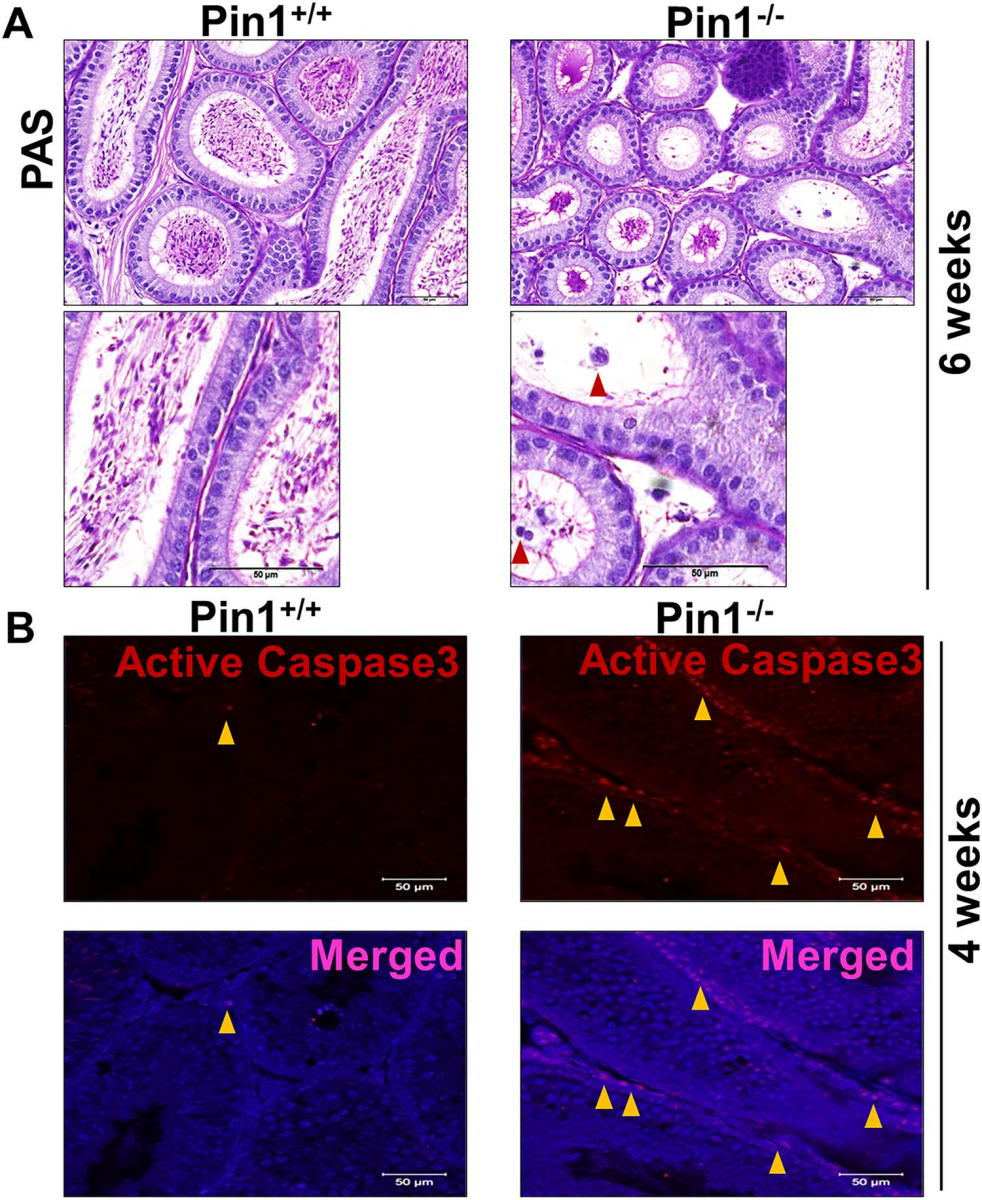
Pin1^−/−^ testis shows signs of compromised immune privilege. (**A**) Epididymides of 6-week-old mice were fixed and sectioned to observe any abnormalities. Red arrows indicate blood cells or abnormal germ cells (scale bar = 50 µM). (**B**) Testis sections were stained with antibody against active caspase 3 (BD Bioscience) to evaluate apoptosis (scale bar = 50 µM). Yellow arrows indicate cells with nuclei immunostained for active-caspase 3 (Cy3-stained).

Upon closer observation of Pin1^−/−^ testes, we found cells that appeared to be blood cells, including particularly distinctive eosinophils (with kidney-shaped lobed nuclei), which are rarely present in the epididymis lumen unless there has been damage or a breach in the BTB (Fig. 3A). Cell debris and eosinophilic inclusions were observed in the tubular lumen of the epididymis (Fig. 3A).

Many genetic mouse models with a testis degeneration phenotype have been shown to have increased levels of germ cell apoptosis. Previous reports inferred from the results of TUNEL assays that there was no increase in apoptosis in Pin1^−/−^ testes^16^. Sertoli cell vacuolation and tubular dysgenesis strongly indicate the probability of mass apoptosis. We opted to evaluate apoptosis using cleaved caspase 3 staining as a marker in frozen sections, as TUNEL assays performed in testicular tissue can yield false results^29^. We observed that Pin1^−/−^ testes showed a markedly high number of tubules containing cleaved caspase 3-positive cells at 4 weeks of age, whereas Pin1 +/+ testis had very few (almost none) cleaved caspase 3-positive cells (Fig. 3B). The presence of blood cells and the increase in cleaved caspase 3 staining indicate compromised immune privilege in the Pin^−/−^ testis, leading to apoptosis of tubular resident cells.

### BTB integrity was disrupted by the absence of Pin1

The BTB is “one of the tightest junctions” in the mammalian body^30^. The BTB in mammalian testes is not like other blood-tissue barriers. It is constituted almost exclusively of specialized junctions between adjacent Sertoli cells near the basement membrane in the seminiferous epithelium of the seminiferous tubule, which is not penetrated by blood vessels, lymph vessels, or nerves^5^.

**Figure 4:**
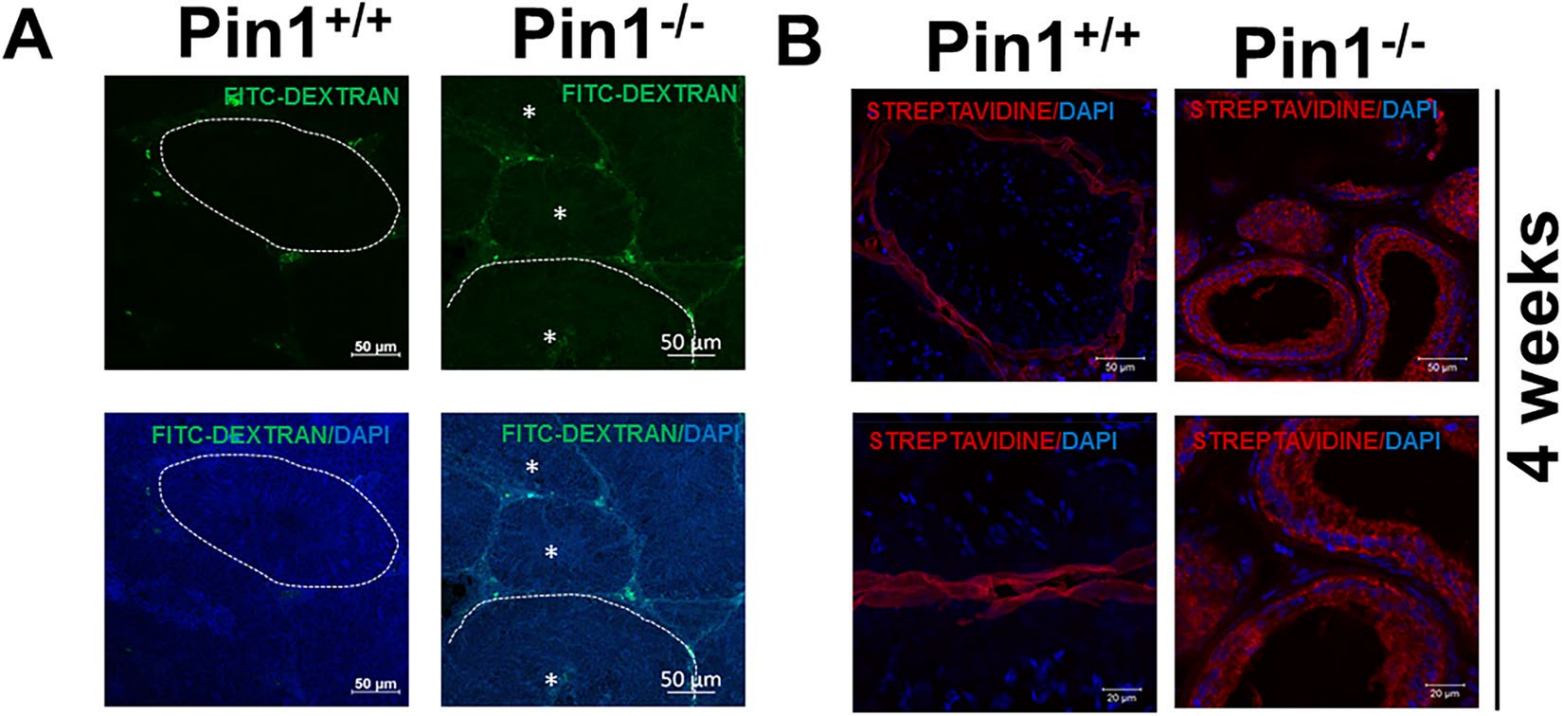
Pin1^−/−^ testes show a BTB defect. (**A**) Testes from mice injected with Dextran-FITC were analyzed for a BTB defect. Mice injected with Dextran-FITC through the jugular vein were sacrificed 2 hr later. Testes were fixed immediately and later paraffin embedded. Sections were cut and analyzed using FITC staining. The whole process was carried out with light protection (scale bar = 50 µM). (**B**) A biotin tracer was injected in the interstitium of the testicular tissue, and samples were collected 30 min post sacrifice to further confirm the BTB defect. CY3-conjugated streptavidin was used to detect the presence of biotin in the adluminal compartment after frozen sectioning (upper panel scale bar = 50 µM and lower panel scale bar = 20 µM).

To test BTB integrity, we injected mice with dextran–FITC using an intravenous route through the jugular vein and collected tissue after 2 hours. This BTB integrity assay is based on the ability of the BTB to block diffusion of a molecular probe, such as dextran-FITC, in the systemic circulation from traversing the BTB. In Pin1^+/+^ testes, FITC fluorescence was observed only in the blood vessels, interstitium, and basal compartment of the tubules. However, FITC fluorescence was present even in the adluminal compartment of Pin1^−/−^ testes (Fig. 4A).

The BTB is not an endothelial cell barrier like the blood brain barrier or the blood placental barrier; rather, it is formed by tight junction complexes between Sertoli cells (Sertoli cell-Sertoli cell tight junctions) and is an interstitial tissue barrier. So, to further confirm the integrity of the barrier, we needed to inject a tracer in the interstitial tissue and observe diffusion. Dextran-FITC is not ideal for this purpose because there is a chance of glucose diffusion. Thus, BTB integrity was evaluated using a biotin tracer to determine whether assembly of the BTB was impaired by Pin1 deletion. The biotin tracer solution was injected into the interstitium of the testes in 4-week-old mice. After 30 min, the animals were euthanized, and the biotin signals in frozen testis sections were detected using fluorescence-conjugated streptavidin and confocal microscopy. In the Pin1^+/+^ male mice, the biotin tracers were restricted to the interstitial space and the basal compartment, whereas the biotin tracer penetrated the adluminal compartment and diffused throughout almost all of the seminiferous tubules of the Pin^−/−^ males (Fig. 4B). These observations suggest that BTB permeability was increased and the formation of the BTB was disturbed in Pin1^−/−^ mice, similar to results described for other models with a dysfunctional BTB^31^.

### Loss of Pin1 causes a reduction in Smad3 protein in the testis

**Figure 5:**
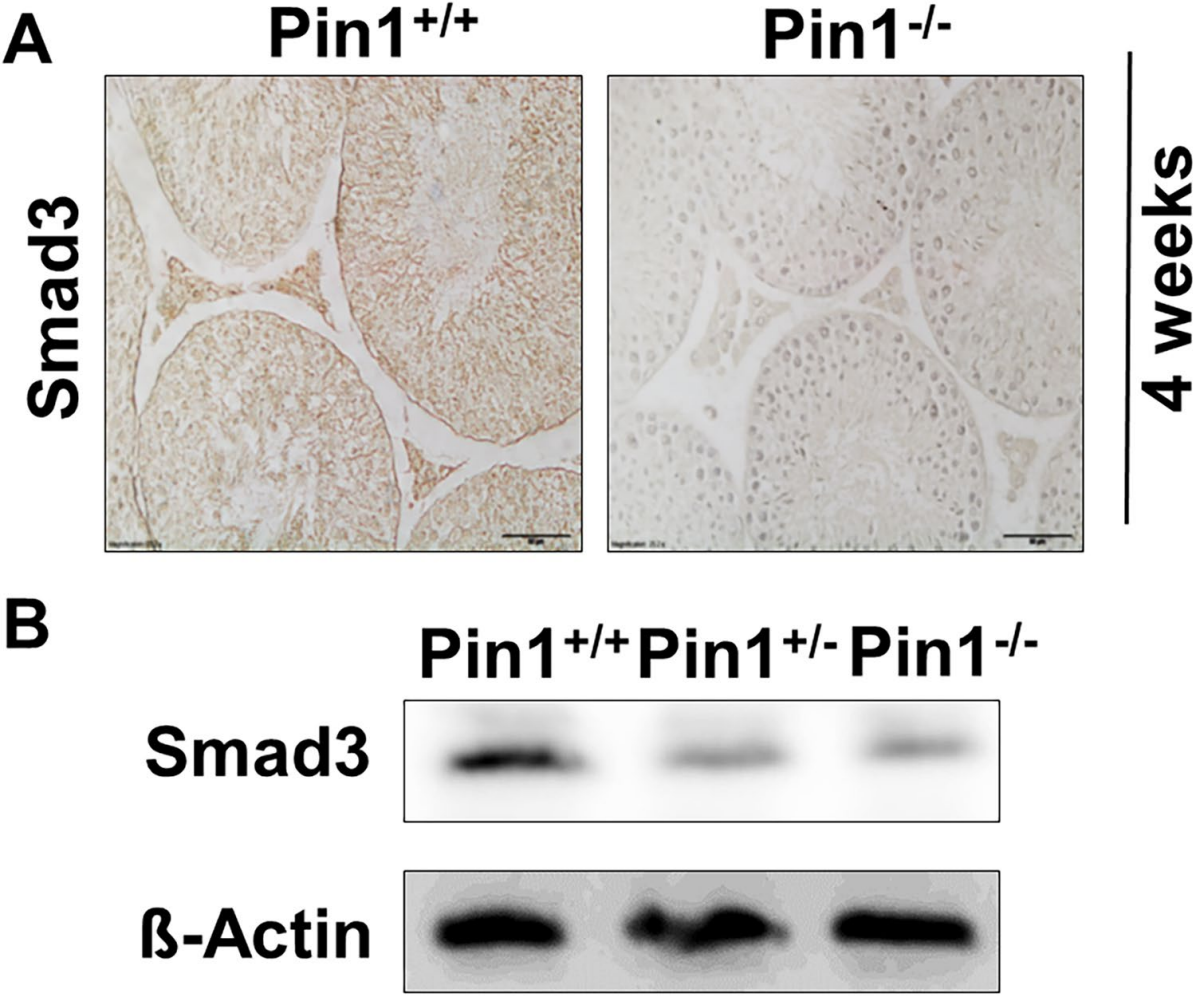
Smad3 expression is decreased in Pin^−/−^ testes. (**A**) Testicular tissues from 4-week-old mice were immunostained for Smad3 protein (scale bar = 50 µM). (**B**) Quantification of Smad3 expression was performed on isolated Sertoli cells from Pin1^+/+^, Pin1^+/−^, and Pin1^−/−^ mice at 4 weeks of age. Freshly isolated cells were lysed in protein lysis buffer immediately after hypotonic shock to remove germ cells. ß-Actin was used as a loading control.

To investigate the underlying mechanism for the observed phenotype of Pin1^−/−^ testes, we investigated several proteins related to testis maturity. Activin signaling is the most important signaling pathway involved in Sertoli cell maturation^32^. SMAD3 is the key mediator of activin-induced Sertoli cell maturation^33^. It was previously reported by us and others that the R-Smad proteins are important substrates of Pin1^20, 34^. Smad3 is directly linked to Sertoli cell maturation^35^. We found that the expression of Smad3 was reduced in Pin1^−/−^ testicular tissue (Fig. 5A) and isolated Sertoli cells (Fig. 5B).

### N-cadherin expression is decreased in Pin−/− testes and unorganized in TM4 cells after Pin1 inhibition

**Figure 6:**
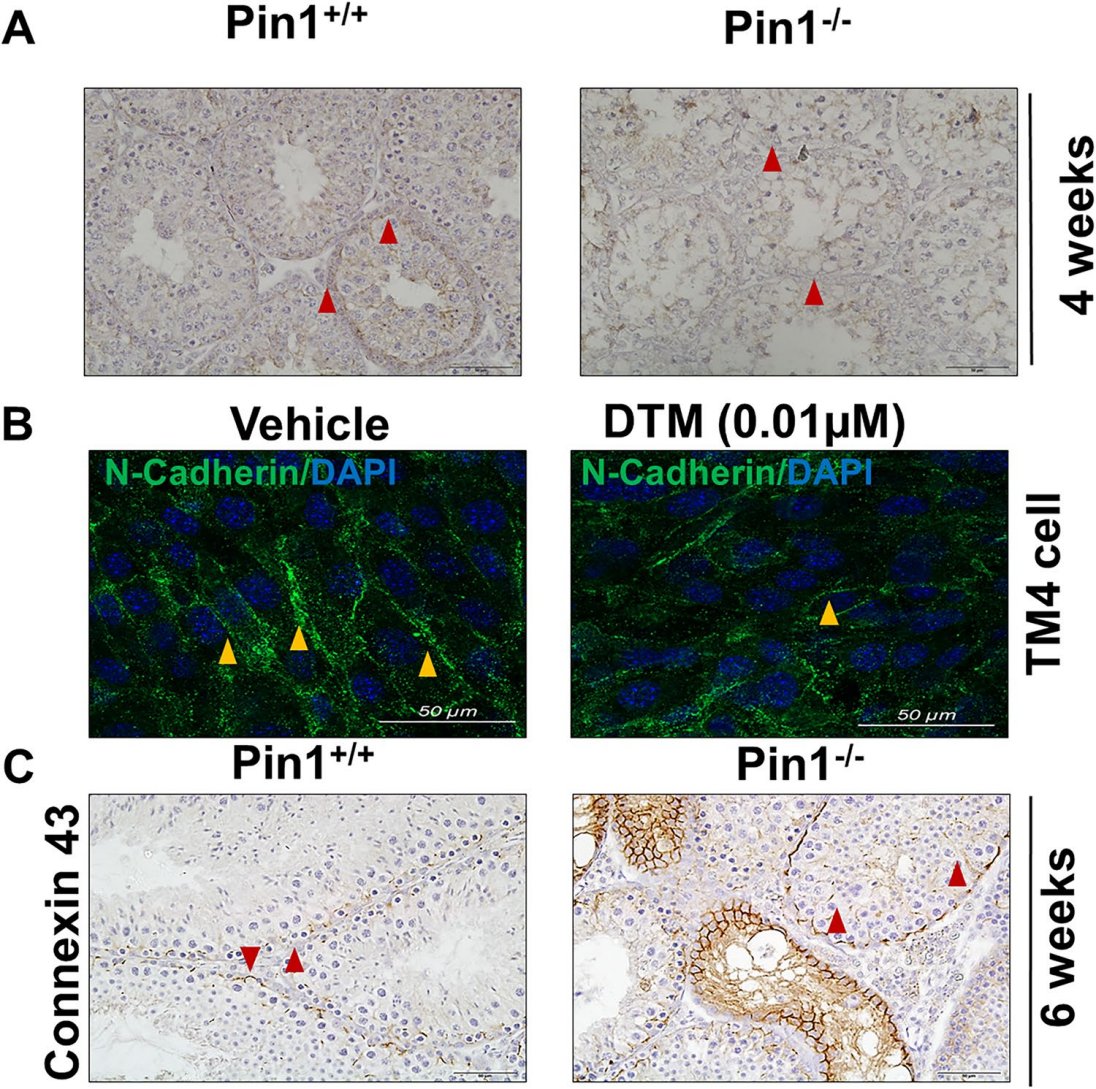
N-cadherin expression is decreased in Pin^−/−^ testes and is unorganized in TM4 cells after Pin1 inhibition. (**A**) Testicular tissue sections from 4-week-old mice were immunostained for N cadherin expression (scale bar = 50 µM). (**B**) Confocal microscopy images of TM4 cells with endogenous Pin1 catalytic function inhibited by DTM (0.01 µM) a known inhibitor for Pin1 activity. Cells were stained for N-cadherin (Alexa-488/ green) and counterstaining with DAPI (scale bar = 50 µM). (**C**) The BTB junction protein Connexin 43 was immunostained in testicular tissue of 6-week-old Pin1^+/+^ and Pin1^−/−^ mice (scale bar = 50 µM).

Smad3 is known to regulate junction protein expression during epithelial to mesenchymal transition in cancer^36^. We examined the expression of N-cadherin (N-Cad; Cdh2), a molecule that contributes to the formation of the BTB and a constituent protein of adherens junctions^37^. A previous report demonstrated that diminished TGF-β-induced cell migration and invasion in the Pin1 knockdown cells correlated with greatly reduced expression of N-cadherin in cancer^38^. Downregulation of N-cadherin was observed in Pin1^−/−^ testes by immune-histofluorescence (Fig. 6A), whereas the expression of ZO-1 remained the same as in control testes (data not shown). Finally, to reinforce these data, we studied N-cadherin expression in TM4 cells treated with the Pin1 catalytic inhibitor DTM (0.01 µM). In the TM4 Sertoli cell line, staining of this intercellular junction component decorated the surface of TM4 cells and showed uniform organization, whereas this pattern was lost upon DTM treatment and revealed disorganized expression (Fig. 6B). Reductions in Pin1 reduce N-Cadherin expression, and inhibition of Pin1 activity results in loss of proper junctional localization of N-Cadherin.

Connexin 43 (Cx43), also known as GJ protein alpha 1 (Gja1), is the predominant testicular gap junction protein located between adjacent Sertoli cells and between Sertoli cells and germ cells and is essential for normal BTB assembly^39^. We examined the expression and location of Cx43. In normal mice, the BTB was formed at approximately 2 weeks of age, and Cx43 was arranged in a regular pattern, which divided the seminiferous tubules into two parts (basal and adluminal compartments) (Fig. 6C, left images). However, these junction proteins displayed aberrant localization and were dispersed over the entire tubules in Pin1^−/−^ mice. In addition, Cx43 expression was significantly increased (Fig. 6C, right images), similar to other mutant model mice with a BTB defect^31^. Together, these observations suggest that Pin1 deletion causes destruction of the BTB integrity because of a dysregulation of the proteins that maintain the cell to cell communication.

### The mRNA expression profile of Sertoli cell maturation markers matched that resulting from reduced Smad 3 dosage, with the exception of Wt1 (Wilms tumor gene) expression

**Figure 7:**
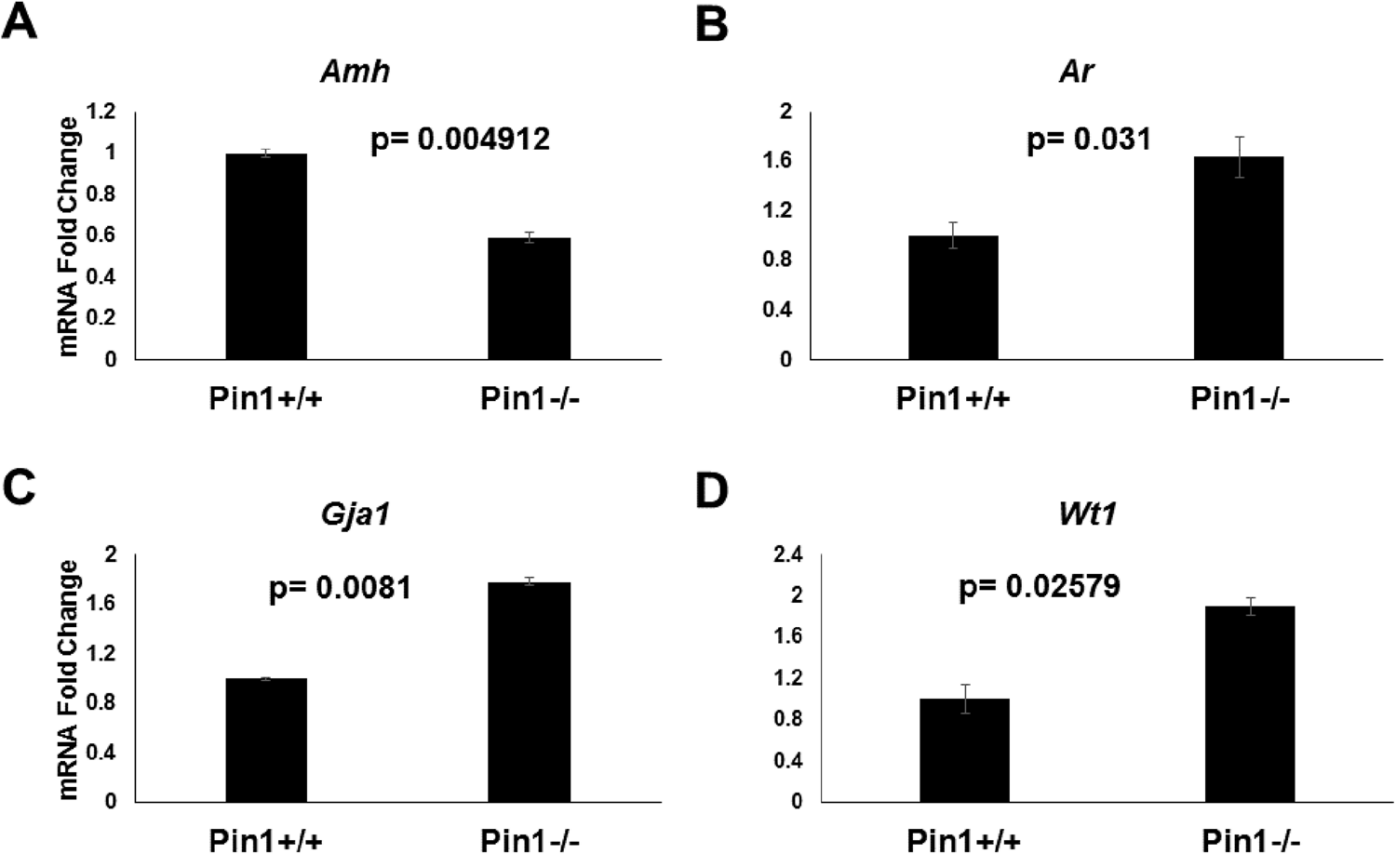
Sertoli cell markers are dysregulated in Pin1^−/−^ testes. (**A**) Amh (antimüllerian hormone) mRNA transcript expression was analyzed in Pin1^+/+^ and Pin1^−/−^ testes. (**B**) Ar (androgen receptor) and (**C**) Gja1/connexin 43 mRNA levels were also analyzed in the same tissue. (**D**) The expression profile of the Sertoli cell-specific marker Wt1 (Wilms tumor) was analyzed by qRT-PCR. RNA from whole snap-frozen testicular tissue was extracted by Trizol digestion after homogenization using a mortar. qRT-PCR was performed using Sybr green reagent in an Applied Biosystems 2500 thermocycler.

We analyzed the Sertoli cell maturation markers in maturing 16-day testis, which correlates with the first wave of spermatogenesis. The measured transcripts associated with Sertoli cell maturation were anti-müllerian hormone (Amh) [down-regulated during maturation^40^] and Gja1 (connexin 43) and androgen receptor (Ar) (up-regulated as Sertoli cells mature)^33^. We found that Amh mRNA showed a greater reduction in Pin^−/−^ testis compared to Pin1^+/+^ (Fig. 7A), whereas Gja1 and Ar both showed greater increases compared to wild type testis (Fig. 7B and C). This mRNA expression profile mimicked the profile of the Smad3^+/−^ testis mRNA expression levels during Sertoli cell maturation^33^.

Differences in germ to Sertoli cell ratios between genotypes were assessed by comparing the expression of Wilms tumor homolog 1 (Wt1), which is a known marker of Sertoli cell maturation, is specifically expressed in Sertoli cells, and supports spermatogenesis. Wt1 is a critical determinant of Sertoli cell fate as dysregulation of this gene can cause reprograming of Sertoli cells to immature Leydig cells^41–44^. Previously, Wt1 was reported not to change with Smad3 dosage^33^ and germ cell ablation^45^. We found that Wt1 was more highly expressed In Pin1^−/−^ testes than in Pin1^+/+^ testes (Fig. 7D). From our data, we deduce that the Sertoli cells of Pin1^−/−^ mice did not have a maturation defect.

## Discussion

Appropriate Sertoli cell organization and development underpin normal adult fertility; thus, identifying how these processes are controlled is relevant to understanding sub-fertility or infertility and disorders of testis development. It is also particularly relevant to the increasing incidence of testicular dysgenesis. Pin1 is strongly expressed in testicular tissue and plays a unique role that cannot be substituted by other PPIases^15^. It is known to be expressed in Sertoli cells^16^, but this is the first time the role of Pin1 in Sertoli cell has been investigated. Pin1 knockout mice display features like testicular dysgenesis syndrome (TDS), where testis just after maturation appears to be similar to wild type testis, but subsequently shows a derangement of seminiferous tubular structure and consequent infertility^17, 28^. Previous studies focused on germ cells as the cause of this dysgenesis. However, most of these studies were based on data derived from testes of mice that were 3 months old or older^39^. We utilized 4- and 6-week-old testes so that the peak period for the initiation of mass tubular dysgenesis could be studied. Based on several observations, it has been hypothesized that TDS arises in fetal life due to the abnormal development of Sertoli and Leydig cells or due to environmental factors that impair the normal function of these cells^13^. Support for this concept comes from the analysis of the contralateral testes of men with testicular cancer, which revealed areas of dysgenesis (poorly formed seminiferous tubules), transformed fetal germ cells, areas with immature or abnormal Sertoli cells lacking germ cells, and foci of Leydig cell hyperplasia^13^. Findings from our study indicate that Pin1^−/−^ mice undergo abnormal Sertoli cell organization caused by dysregulation of the proteins that control cell to cell communication (Fig. 6). This might be the driving force behind the global gonadal dysgenesis observed later during aging (Figs 1 and 2).

Previous studies have shown that primordial germ cells (PGCs) in Pin1^−/−^ mice have a prolonged cell cycle, which translates into fewer cell divisions^28^. At the same time, quantification revealed that the percentage of Pin1^−/−^ PGCs in mitosis (6.4%) was not significantly different from that of wild type (5.8%), indicating that mammalian Pin1-deficient PGCs progressed through mitosis^27^. These findings cannot sufficiently explain the phenotype of Pin1^−/−^ testis. A reduction in only germ cell number cannot explain the normal features seen in younger Pin^−/−^ mice or the irregularly spread gradual seminiferous tubule degeneration seen with aging (Fig. 1A and B). We found that, at the same time that dysgenesis of the seminiferous tubules in Pin^−/−^ mice is initiated, the BTB fails (Figs 2 and 3). This coincides with ongoing mature sperm production in other tubules (Fig. 1). Abnormal permeability of the BTB (Fig. 4) can result in the release of germ cells into the bloodstream that leads to the mounting of an autoimmune response and the production of anti-sperm antibodies, which subsequently cause apoptosis. Based on our results, we propose that disruption of the BTB is the first step in the degenerative process occurring in Pin1^−/−^ testes, which ultimately leads to apoptosis of germ cells (Fig. 2) and gradual atrophy due to mass dysgenesis.

Activin and its mediators, the R-Smads, are the most important group of proteins in Sertoli cell maturation^32^. Testosterone, together with TNFα and TGF-β, also promotes the junction integrity of the BTB^46^. R-Smads are the main mediators of these signaling pathways. Among R-Smads, Smad 3 has a very significant role in Sertoli cell maturation^33^. Pin1 plays a critically important role in the early stages of Sertoli cell maturation to maintain the BTB. Downregulation of Smad3 to a certain threshold results in normal juvenile testis growth. A study relating Smad3 level to the timing of the first wave of spermatogenesis identified the Smad3^+/−^ mouse as a model of peripheral precocious puberty and the Smad3^−/−^ mouse as one of delayed Sertoli cell development^21^. As the absence of Pin1 results in reduced Smad3 expression, our data indicate that isomerization of Smad3 at these stages might be an integral part of the process (Fig. 5). The Pin1^−/−^ mouse showed no defects in Sertoli cell maturation (Fig. 7), but did show defects in Sertoli cell-to-cell contact (Figs 4, 5 and 6). However, unlike Pin^−/−^ mice, Smad3^−/−^ mice do not become infertile^33^, which indicates that the reduced germ cell numbers^16^ seen in the Pin1^−/−^ mice further aggravate the destruction of the BTB junction. A comparison of Sertoli cell differentiation in Pin1^+/−^ and Pin1^−/−^ mice to the well-characterized Smad3^+/−^ and Smad3^−/−^ mouse models of Sertoli cell defects suggested that Sertoli cells are the primary cell type affected by Pin1 dosage.

Smad2 and Smad3 proteins are differentially regulated in the testis^35^. Pin1 binds both Smad2 and Smad3^47^. This might be an important topic for further study in testis physiology. In the first two neonatal weeks, Sertoli cells undergo a polarity establishment process for tight junction formation between cells, during which apical–basal polarity is formed. Consequently, Sertoli cells adopt a more epithelial phenotype in the mesenchymal–epithelial balance during this period^42^.

Wt1 also regulates the mesenchymal–epithelial balance of Sertoli cells through E-cadherin^44, 48, 49^. Pin1^−/−^ testes showed an upregulation of Wt1 mRNA (Fig. 7) and a downregulation of the N-Cadherin protein (Fig. 6), which might cause an imbalance of the epithelium to mesenchymal ratio. The N-cadherin–β-catenin complex at the basal ES is also crucial for BTB maintenance. Previously, we reported that Pin1 regulates the export of β-catenin from the nucleus^50^, which might affect β-catenin localized at the membrane. Thus, we propose that Pin1 can affect the mesenchymal–epithelial balance in Sertoli cells via N-cadherin, β-catenin^50^, and other known and unknown target proteins of Wt1. However, the precise role of Pin1 in the developing testis needs to be further studied, especially in the context of the supporting Leydig and Sertoli cells.

Endocrine-disrupting chemicals are linked to delayed puberty and impaired androgen actions in boys^51, 52^. Studies using rats support these human data, with endocrine disruptors exerting broad effects on the developing testis, including changes in Sertoli cell proliferation, maturation, and androgen responsiveness, with the developing testes being more sensitive than the adult testes^51^. The Pin1^−/−^ mouse model is a phenocopy of abnormal BTB function. Pin1-mediated regulation of junction proteins (N-cadherin and β-catenin) therefore offers the opportunity to identify new targets for investigations into reproductive toxicology and altered pubertal development, as well as the development of potential male contraceptives. Our data identified a unique role of Pin1 in post-mitotic Sertoli cells as a crucial factor in BTB maintenance.

## Electronic supplementary material

Dataset 1
