# Supplementary material for: Blood-testis barrier integrity depends on Pin1 expression in Sertoli cells
Source: Sci Rep. 2017 Aug 1;7:6977. doi: 10.1038/s41598-017-07229-1 (PMC5539286; doi:10.1038/s41598-017-07229-1)
Supplement: Supplementary file 1 — Dataset 1 [file 41598_2017_7229_MOESM1_ESM.doc]

**Blood-testis barrier integrity depends on Pin1 expression in Sertoli cells**

**Rabia Islam, Heein Yoon, Bong-soo Kim, Han-sol Bae, Hye-rim Shin, Woo-Jin Kim, Won-joon Yoon, Yun-Sil Lee, Kyung Mi Woo, Jeong-Hwa Baek and Hyun-Mo Ryoo**

**Department of Molecular Genetics, School of Dentistry and Dental Research Institute, Seoul National University, Seoul, 110-749, Korea**

**Correspondence to:**

**Hyun-Mo Ryoo**

**Department of Molecular Genetics & Dental Pharmacology**

**School of Dentistry, Seoul National University**

**1 Gwanak-ro, Gwanak-gu, Seoul 08826, Republic of Korea**

**E-mail: hmryoo@snu.ac.kr**

**Tel: +82-2-880-2320 Fax: +82-2-741-3103**


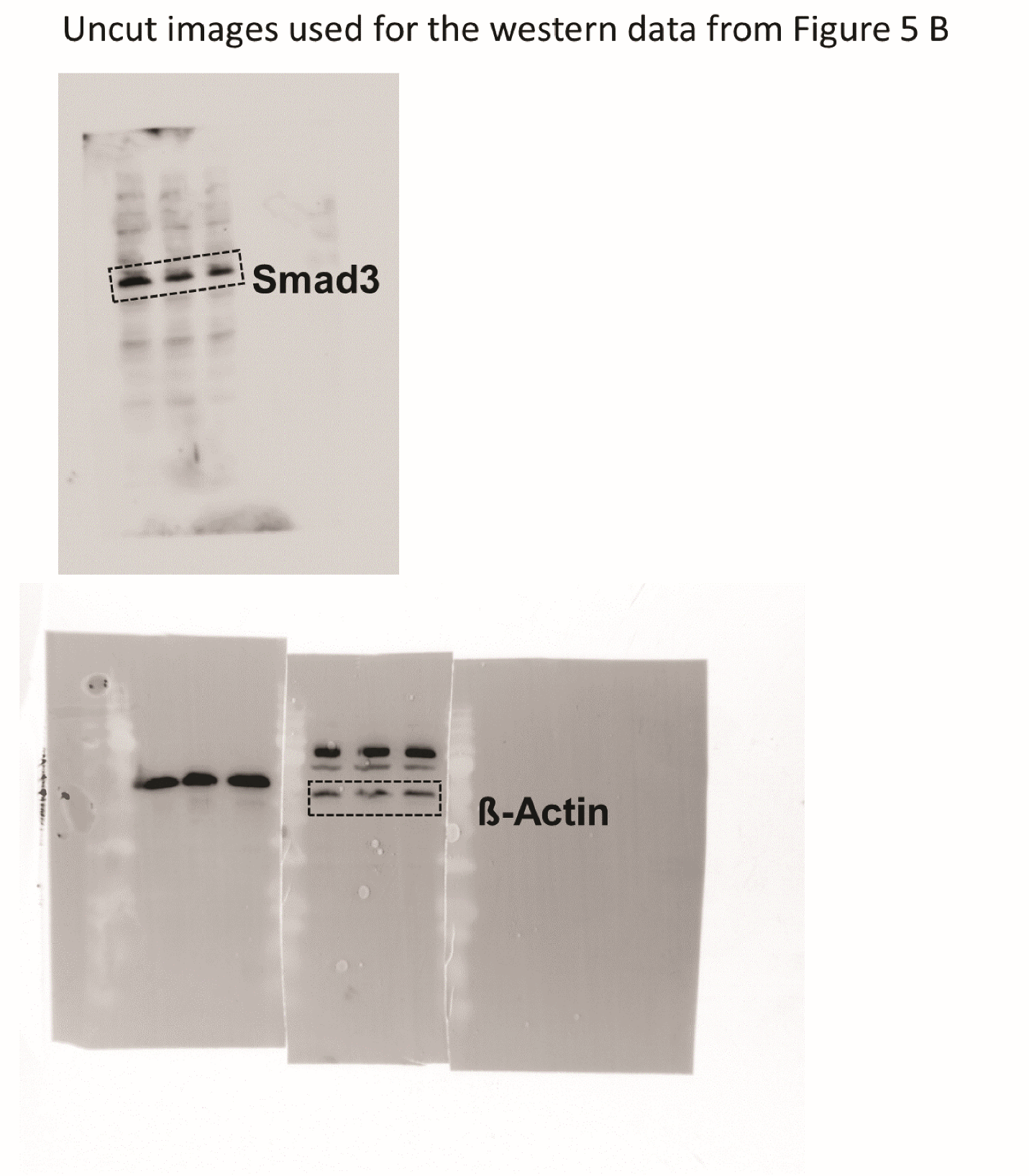


Supplementary Figure 1- Uncut western data used for Figure 5B


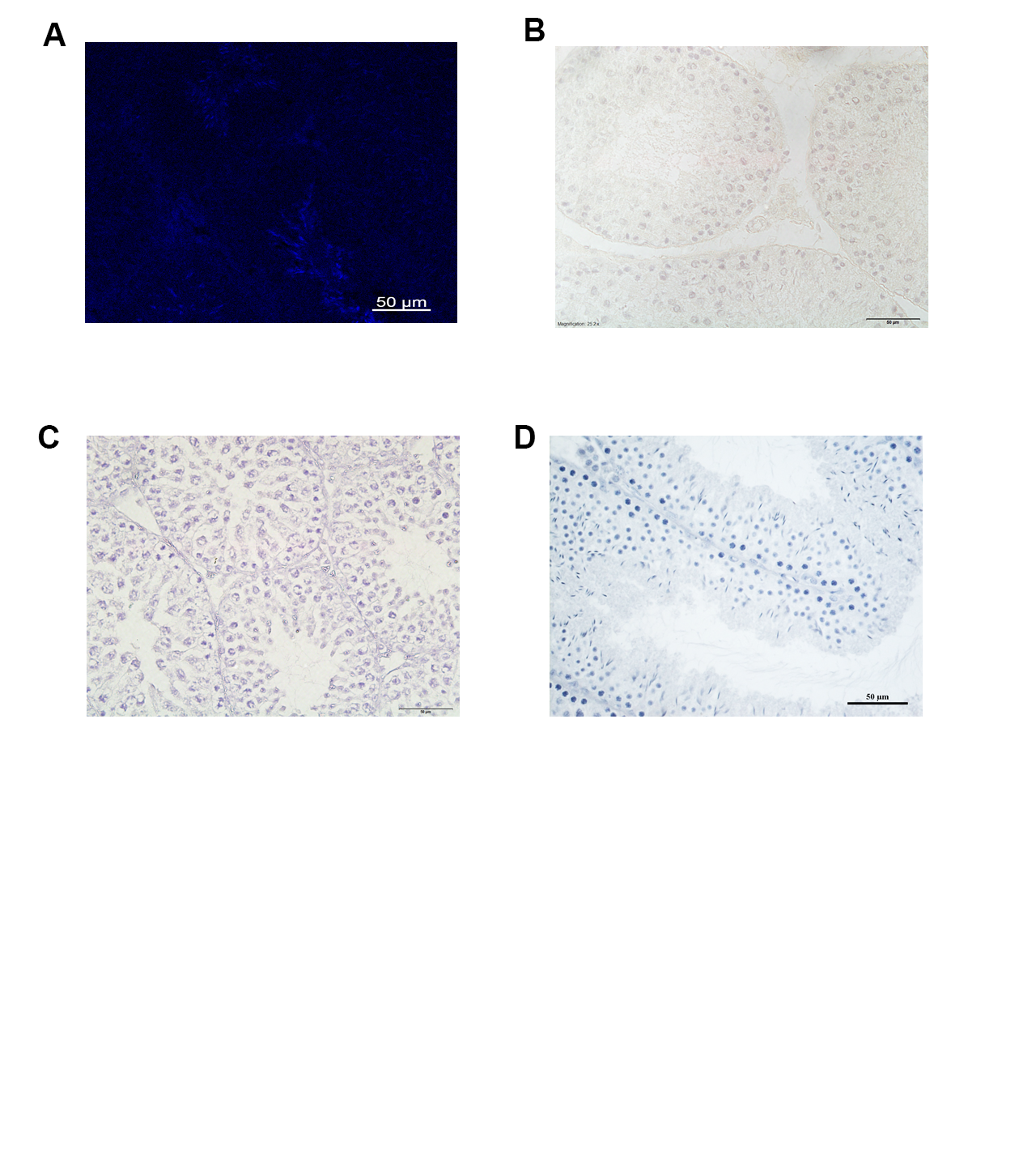


Supplementary Figure 2- Negative control IgG images for (A) Figure 4 FITC-Dextran assay, (B) Smad3 Immunohistochemistry (Figure 5A), N- Cadherin Immunohistochemistry (Figure 6A) and Cx43 Immunohistochemistry (Figure 6C) on Pin1^+/+^ testes tissue.
